# HIV pre‐exposure prophylaxis for adolescent girls and young women in Africa: from efficacy trials to delivery

**DOI:** 10.1002/jia2.25298

**Published:** 2019-07-22

**Authors:** Connie L Celum, Sinead Delany‐Moretlwe, Jared M Baeten, Ariane van der Straten, Sybil Hosek, Elizabeth A Bukusi, Margaret McConnell, Ruanne V Barnabas, Linda‐Gail Bekker

**Affiliations:** ^1^ Department of Global Health University of Washington Seattle WA USA; ^2^ Department of Medicine University of Washington Seattle WA USA; ^3^ Department of Epidemiology University of Washington Seattle WA USA; ^4^ Wits Reproductive Health and HIV Institute University of Witswatersrand Johannesburg South Africa; ^5^ RTI International Women's Global Health Imperative (WGHI) Program San Francisco CA USA; ^6^ Department of Psychiatry Stroger Hospital Chicago IL USA; ^7^ Kenya Medical Research Institute Nairobi Kenya; ^8^ Departments of Obstetrics‐Gynecology University of Washington Seattle WA USA; ^9^ Department of Global Health and Population Harvard T.H. Chan School of Public Health Boston MA USA; ^10^ Desmond Tutu HIV Centre University of Cape Town Cape Town South Africa

**Keywords:** HIV prevention, pre‐exposure prophylaxis, adolescents, young women, Africa

## Abstract

**Introduction:**

Adolescent girls and young women (AGYW) in Africa have high HIV incidence despite scale‐up of HIV testing and HIV treatment. Placebo‐controlled trials of tenofovir‐based pre‐exposure prophylaxi (PrEP) in diverse populations demonstrated that PrEP works with close to 100% effectiveness if taken with high, but not perfect, adherence. Divergent efficacy estimates among African AGYW led to demonstration and implementation projects to better understand motivations for HIV prevention, uptake, adherence and persistence to PrEP. To inform PrEP programmes, the design and initial findings from PrEP demonstration projects for AGYW are reviewed.

**Discussion:**

Early lessons from PrEP implementation projects among young African women include: (1) awareness and demand creation with positive messaging about the benefits of PrEP are critical to motivate AGYW to consider this novel prevention technology and to foster awareness among peers, partners, parents and guardians to support AGYW's effective PrEP use; (2) PrEP initiation is high in projects that are integrating PrEP into youth‐friendly clinics, family planning clinics and mobile clinics; (3) young African women at risk are initiating PrEP, based on behavioural characteristics, history of intimate partner violence, depression and 30% prevalence of chlamydia and/or gonorrhoea; (4) provision of youth‐friendly PrEP delivery programmes that integrate reproductive health services, including contraception and the diagnosis and treatment of sexually transmitted infections, increase health impact; (5) messages that emphasize the necessity for high adherence while at potential risk of HIV exposure and support strategies that addresses AGYW's adherence challenges are essential; and, (6) a substantial proportion of AGYW do not persist with PrEP, and strategies are needed to help AGYW assess their ongoing need, motivation and challenges with persisting with PrEP.

**Conclusions:**

PrEP is feasible to implement in integrated reproductive health service delivery models to reach African AGYW. While PrEP demonstration projects indicate that women with behavioural risks and high rates of sexually transmitted diseases are initiating PrEP; effective strategies to support AGYW's adherence and persistence with PrEP are needed. Lessons learned from oral PrEP delivery, a novel first generation HIV prevention product, are relevant to longer‐acting and less adherence‐dependent strategies which are currently in clinical trials.

## 1 Introduction

Adolescent girls and young women (AGYW) in Africa account for approximately 25% of new HIV infections globally, and have large unmet needs for HIV prevention 1. African AGYW experienced high HIV incidence despite monthly counselling and prevention services in recent biomedical HIV prevention trials 2. Excitement about pre‐exposure prophylaxis (PrEP), a novel approach to HIV prevention, coalesced when trials demonstrated estimates of HIV protection of >90% in women and men 3. However, in PrEP efficacy trials, protection ranged from 0 to 75% in different populations 4, 5, 6, 7, 8, 9 with the variation largely explained by differences in participants’ level of adherence, based on retrospective testing of drug levels. Indeed, PrEP is a user‐controlled method, highlighting the strong behavioural component to PrEP uptake, adherence and persistence, which was most apparent among African AGYW. This commentary synthesizes evidence and expert opinion about PrEP efficacy and implementation for African AGYW, spanning from efficacy trials to emerging data from multiple demonstration projects.

## 2 Discussion

### 2.1 Challenges with oral PrEP among young African women

Protection was 72% among young, high‐risk women in subgroup analyses of female partners in HIV serodiscordant couples in the Partners PrEP study where adherence was 80% 10. In contrast, in the VOICE and FEM‐PrEP trials, young women were not protected, most of whom had a partner of unknown HIV status, and among whom only 25% had detectable tenofovir levels 4, 5. In VOICE, young women (<25 years) were at greatest risk for HIV infection and less likely to adhere. Nevertheless, a subsequent meta‐analysis of all PrEP trials including women estimated 61% PrEP efficacy among women with > 75% adherence based on drug levels 11.

The results of the VOICE and FEM‐PrEP trials led to concerns that young African women did not recognize their risk, would lack motivation or be unable to adhere to daily pill‐taking for HIV prevention. Notably, the quantitative measure of risk perception in these trials was based on a single question, which may be insufficient for understanding how women viewed their risk. In contrast, in‐depth interviews indicated that women often recognized their risk 12. Socio‐behavioural research during and following the VOICE and FEM‐PrEP trials found that participants had altruistic motivations for joining the studies but were balancing this against the practical realities of their daily lives. In the African context where access to quality care and resources is limited, participants desired access to non‐judgemental, confidential clinical services, counselling support, and contraception, HIV and sexually transmitted infection (STI) testing and treatment which were provided by the trial sites 13, 14. Small study reimbursements (<$15) helped participants meet transport costs and other needs 14, 15, 16.

Women faced many challenges in these studies, including their concerns about receiving a placebo or an investigational drug of unknown efficacy and potential side effects 17, 18, 19, 20. “Present bias” is the tendency to disproproportionately focus on rewards in the present to the detriment of achieving desired long‐term outcomes 21, 22, 23, and is a strong motivator in AGYW, which could have discouraged committed product usage. Qualitative research suggests that rumours and peers’ comments about not using their study products influenced participants in terms of their adherence and willingness to disclose actual product use 24. Women often experienced low social support for joining the trials or for using a female‐controlled method of HIV prevention, and had to balance their motivation for HIV prevention with fear of their partner's reactions and possible violence. Indeed, women's desire to preserve their relationship and trust their partner may weigh more in their lives than their risk of acquiring HIV and prevention considerations 19, 25. Intimate partner violence (IPV) has since been shown to have been associated with non‐adherence 26, 27, 28. HIV and stigma associated with antiretrovirals were also barriers to product use. AGYW reported having limited private storage space, fears of inadvertent disclosure to family and partners, and subsequent misperceptions about HIV serostatus, contributing to poor adherence among those who tried to conceal product use 29. Adherence to a daily product was particularly challenging for young women where executive functions and organizational skills are still developing 30, 31, 32. Executive function has been shown to play a role in adolescent medication adherence for chronic illnesses as well as preventive care 33, 34, 35, 36, 37.

Acceptability research conducted since VOICE and FEM‐PrEP indicate that women desire low burden prevention strategies that are compatible with their lifestyles and provide peace of mind 38, 39. Indeed, a daily pill regimen can be both logistically and emotionally burdensome (as it may remind women about HIV or IPV) 16. When offered HIV prevention alternatives through discrete choice experiments which assess hypothetical preferences and trade‐offs between them, women prefer longer‐acting and more adherence “forgiving” products, supporting the development of a range of PrEP delivery modalities – ring, injectable, implantable – from which young women can choose 38, 40, 41. In parallel with development of formulations with less frequent dosing, it is important to learn about delivery and use of oral PrEP, a vanguard product.

### 2.2 Lessons about PrEP effectiveness from other populations

Open‐label PrEP studies have demonstrated that risk perception and HIV prevention motivation might be less of a challenge than initially anticipated. Significantly higher effectiveness than efficacy was observed in open‐label studies, including the PROUD study (immediate vs delayed open‐label PrEP among men who have sex with men (MSM) attending sexual health clinics in the UK) 42 and HIV serodiscordant couples in the Partners Demo Project 43, 44, which provided time‐limited PrEP for HIV‐uninfected partners until the HIV‐positive partner was virally suppressed on antiretroviral therapy (ART). Similarly, effectiveness of the first longer acting PrEP product – the dapivirine ring – was higher in an open‐label extension study than in the placebo‐controlled efficacy trials 45.

Studies specific to adolescents and youth have provided useful and encouraging data. In the HPTN 067/ADAPT trial of daily, fixed intermittent or event‐driven dosing among young women in Cape Town, adherence to daily PrEP was 75% and daily dosing provided the highest coverage of sex acts 46. A US‐based study of oral PrEP among MSM ages 15 to 17 (Adolescent Trials Network [ATN] 113) demonstrated that the majority had intracellular tenofovir‐diphosphate (TFV‐DP) levels commensurate with HIV protection (>700 fmol TFV‐DP/punch) over the first three months 47, which decreased over the second six months when visits became quarterly. Similarly, the PlusPills study conducted in South Africa among adolescent boys and girls ages 15 to 19, found that PrEP was safe, acceptable and well‐tolerated in this age group, but that adherence dropped in the second half of the study when visits were spaced quarterly instead of monthly 47. In summary, open label PrEP studies have demonstrated that risk perception, HIV prevention motivation, and time‐bounded use of PrEP might be less of a challenge than initially anticipated, although strategies are needed to support PrEP adherence and persistence.

### 2.3 PrEP implementation for African young women: early lessons

In 2016, WHO recommended that PrEP be targeted to persons at “substantial” risk for HIV, defined as an annual HIV incidence of 3% or higher without PrEP 48, which includes AGYW in high burden geographies in Africa. Initial PrEP demonstration projects for African young women focused on supporting and studying adherence. Notably, the goal for oral PrEP use should not be perfect adherence but “prevention effective adherence” with high adherence during periods of risk 49.

A number of PrEP implementation projects for African young women are studying PrEP uptake, adherence and persistence, and some projects will evaluate impact using a counterfactual estimate of HIV incidence without PrEP (Table 1). These projects have highlighted the challenges of implementing a novel prevention intervention prior to national guidelines in African countries, and thus in advance of provider training, with limited PrEP awareness and availability.

**Table 1:** Pre‐exposure prophylaxis (PrEP) implementation research projects in African young women

Study name and clintrials.gov number	Population	N	Primary objectives
PlusPills NCT03142256	150 men and women 15 to 19 years, Soweto and Cape Town, South Africa	150	PrEP uptake (i.e. acceptance and initiation) and persistence (i.e. continuation)
EMPOWER South African National Clinical Trials 4353	Young women 16 to 24 years; Johannesburg South Africa and Mwanza, Tanzania	431	Effect of empowerment clubs on PrEP uptake and persistence
HPTN 082 NCT02732730	427 women 16 to 21 years in Cape Town and Johannesburg, South Africa, and Harare, Zimbabwe	427	PrEP uptake, effect of drug level feedback on PrEP adherence, and modelled impact compared to a counterfactual HIV incidence estimate
3P (Partners, Perception, Pills) NCT03142256	200 women 16 to 21 years in Cape Town, South Africa	200	Effect of incentives conditioned on adherence (i.e. objectively measured with drug levels) on subsequent PrEP adherence and persistence
POWER NCT03490058	1504 women 16 to 21 years in:Cape Town and Johannesburg, South Africa, and Kisumu, Kenya	1504	PrEP delivery models (mobile van, youth friendly clinic, family planning clinics) and cost‐effectiveness
Community PrEP NCT03977181	Young women 16 to 25 years in Buffalo City, Eastern Cape Province, South Africa	640	PrEP uptake, persistence and community models of delivery to promote persistence and adherence

### 2.4 Demand creation for PrEP

The need for awareness and demand creation became clear early after launching PrEP demonstration projects for young African women, a population that typically accesses health services primarily for contraception. In the case of PrEP, demand creation involves clear, concise and compelling descriptions about PrEP and why women should be interested in it, especially in the context where there is very limited precedent for taking a pill purely for prevention, as most African AGYW use injectable rather than oral contraception. Importantly, antiretroviral use continues to have substantial stigma and AGYW consistently report concerns that others will think that they are HIV positive. Effective demand creation for PrEP creates awareness and motivates persons at risk of HIV to formulate their sexual health goals and motivations for HIV prevention.

Qualitative and ethnographic research was conducted to develop a demand creation strategy for PrEP among young women for the 3P (Partners, Perceptions and Pills) project in Cape Town, and found that pills are perceived as being for treatment rather than prevention and that emphasizing the positive benefits of PrEP in increasing confidence and empowerment would motivate AGYW to consider PrEP 25. Feedback from young women in focus group discussions during development of a video and print materials recommended that demand creation materials show strong, aspirational and stylish women, message the positive benefits of PrEP in terms of young women's empowerment, and include images of men 50. Demand creation and communication materials about PrEP need to avoid perceptions that PrEP is only for women, and implications that women are responsible for HIV prevention. While 72% of women who viewed the brief motivational video expressed strong interested in PrEP, a minority enrolled soon after viewing the video and those who enrolled and initiated PrEP in the 3P study often needed to hear about PrEP from community outreach workers, community events, peers, and parents 50. AGYW reported that it was difficult to be both a PrEP user and advocate if community, neighbours and significant others were unaware of it 51.

Despite the need for greater community awareness about PrEP, these projects have demonstrated that there is demand for PrEP when AGYW are educated about it, as indicated by >90% uptake in HPTN 082 (Table 2) 52. Young women who are initiating PrEP in these demonstration projects are at risk, as measured by the very high rates of depression symptoms (almost 50%), history of IPV (20% to 50%), and 30% prevalence of gonorrhoea and chlamydia, most of which was asymptomatic 53. These high rates of curable STIs in AGYW initiating PrEP highlight that syndromic case management is inadequate and needs to be replaced with etiologic STI testing, the cost of which has been a barrier to implementation. STI testing can be part of PrEP demand creation for AGYW, inform their risk perception and need for PrEP, and STI treatment can avoid adverse impacts on their fertility.

**Table 2:** Findings from pre‐exposure prophylaxis (PrEP) implementation projects in African young women

Observations from PrEP demonstration and delivery projects	Supporting data from PrEP projects
Demand creation is needed, both prior to and after national guidelines and wider PrEP availability and knowledge about PrEP exist	High interest in PrEP after 90 second motivational video in Cape Town, supplemented with other educational and recruitment strategies 76
AGYW at risk of HIV are initiating PrEP with high uptake	90% to 95% PrEP uptake in EMPOWER, POWER 55, 77 and HPTN 082 50, 55 among women, as indicated by high rates of IPV and STIs
STI prevalence are high among AGYW initiating PrEP	30% prevalence of chlamydia and/or gonorrhoea in EMPOWER, POWER 78 and HPTN 082 53
Importance of PrEP education among influencers of young women (e.g. parents, partners) as they act often as detractors or supporters of PrEP use	*We would meet with other participants and encourage each other during the adherence clubs that we did, we would encourage each other to take PrEP*. HPTN 082 participant *I like the adherence clubs, because we will be learning from each other, everyone will be talking about their experiences in taking PrEP. So if you have some things that you hear in the neighbourhood that used to hurt you, and you hear it from other people, you will feel relieved*. HPTN 082 participant*He [my partner] can even send me a WhatsApp message and ask whether I haven't forgotten to take my pills. Then I would say that I haven't forgotten I will take them. And maybe we are messaging each other and its ten past eight, and then I quickly get up and take them. So he also reminds me sometimes*. HPTN 082 participant
Need to make PrEP access convenient and evaluate the feasibility of community‐based delivery (i.e. existing points of contact with young women such as hairdressers, support groups, adherence clubs)	*I would prefer to access PrEP at a nearby place – than having to travel a distance. Because sometimes you don't have taxi fare, so you end up delaying collection for another day. Or leave it completely…* POWER participant

AGYW, Adolescent girls and young women; IPV, intimate partner violence; STI, sexually transmitted infection.

### 2.5 PrEP adherence and persistence

PrEP demonstration projects among African AGYW indicate the early drop off rates in the first few months after PrEP start are approximately 50%, with about 20% of AGYW restarting PrEP within six months in the POWER study 54. Qualitative research is exploring whether this early stopping is related to AGYW experimenting with a novel idea, concerns about PrEP side effects, not liking or being able to take a pill a day, or reassessment of their sexual health goals. Encouragingly, several demonstration projects have shown higher adherence than in VOICE and FEM‐PrEP based on drug levels; at the three month visit in HPTN 082 intracellular TFV‐DP was detected in 84% of AGYW and 25% had high TFV‐DP levels 52, 55 and in the 3P study, 99% of participants had detectable tenofovir and 50% had high TFV‐DP levels at three months 56.

Young persons are likely to need more PrEP adherence support and more frequent contact with health providers for effective PrEP use and persistence. It is important to build in flexibility into PrEP programs for refill timing, as AGYW may not use PrEP daily and may not come for refills until they are out of pills. One strategy to foster PrEP persistence is to integrate PrEP delivery and refills with other reproductive health services (e.g. every three month injectable contraception and STI testing), making clinic visits more salient and efficient.

Peer support is also important and can be fostered through PrEP clubs, which were well‐attended and valued by two‐thirds of participants in HPTN 082 52. Peer support has been shown to improve adherence in outpatients starting ART 57, and adolescents 58. Group‐based interventions for youth offer an effective way to implement intervention content while providing social support from peers, given the importance of peer opinion during adolescence and evidence that peer norms 59, 60, 61 are important in shaping adolescent behavior 62. Adherence support clubs were pioneered in the FACTS 001 trial 63, 64, were found to be feasible and acceptable to participants and staff, and were identified spontaneously by participants during in‐depth interviews as an important source of adherence support 65. In HPTN 082, adherence clubs were incorporated into the standard adherence package that all participants received, were reported to be highly acceptable and rewarding, and club attendance was associated with higher adherence at three months (>700 fmol/punch TFV‐DP per punch) 52. In contrast, in the EMPOWER study which randomized participants to clubs that included a four‐session empowerment curriculum or standard adherence support, clubs did not translate into additional benefits for PrEP persistence, although they were viewed as a valuable source of peer support 66. The Community PrEP Study in the Eastern Cape (Table 1) is testing adherence clubs in a rural setting compared to clinic‐based drug pick ups. Alternate strategies to promote peer support through virtual clubs (e.g. WhatsApp groups) have the potential to overcome the logistical barriers of in‐person meetings, and warrant further investigation.

PrEP requires attention to detail, organizational skills, and quarterly clinic visits by a young, healthy person. This may be particularly difficult for adolescents with many other salient challenges, including poverty which may impose a cognitive cost that crowds out the attention needed to focus on daily prevention activities which have diffuse and long‐term rewards 67. Small incentives have been shown to improve adherence 68, and $15 cash incentives conditioned on TFV‐DP levels are being evaluated in the 3P project to determine whether incentives are effective to focus adolescents’ attention on immediate rewards, support pill‐taking habit formation, and help overcome potential cognitive biases that make prevention behaviours particularly challenging 69, 70.

## 3 Conclusions

PrEP works, works for women, works when taken during periods of HIV exposure, and offers powerful protection for women who take PrEP. It is useful to consider the lessons from PrEP demonstration projects for African AGYW. Demand creation efforts have underscored the need for positive framing about PrEP that include empowerment messages to create interest, as well as the need for broader community awareness and support to overcome the unfamiliarity with PrEP and to reduce stigma associated with using antiretrovirals for prevention. From a clinical perspective, PrEP can be delivered in simple ways, even through peers as has been demonstrated in Thailand 71. One of the greatest lessons from PrEP projects to date is how little is necessary to deliver it, and that only a subset of users may need more adherence support or frequent contact. Risk assessment and PrEP decision tools need to be evaluated as ways to operationalize prevention effective adherence, reduce provider burden and to augment provider counselling. Simpler models of PrEP delivery are being evaluated, including community‐based refills and use of self‐testing for HIV to expedite visits. Adherence clubs are being evaluated as a strategy to support PrEP persistence, similar to the model of community ART clubs have been demonstrated to be very acceptable and improve ART persistence and adherence among those living with HIV 72.

An important tension with simple, parsimonious PrEP delivery for African AGYW is that they have high rates of depression, IPV, and STIs, and ideally PrEP programmes should include wrap‐around services for mental health and STIs. If PrEP programs are not able to address them directly, they must be prepared for them and have adequate referral sources in place. Given the remarkably high rates of asymptomatic curable bacterial STIs in young African women initiating PrEP, syndromic STI case management is inadequate and needs to be replaced with sensitive and affordable STI testing.

Health economic modelling is needed to define the minimal level of PrEP use at the individual level (based on their “seasons of risk”) and at a population level to have a public health impact. Given constrained resources, cost analyses and time‐motion studies are needed of different facility‐based, mobile and community‐based PrEP delivery models. To estimate the health benefit of PrEP, prevention effective adherence will need to be balanced against the risk of HIV acquisition and the impact of PrEP delivery on health resources, which are the opportunity costs of delivering PrEP. Within a streamlined PrEP delivery model in a high HIV burden setting, PrEP use among persons at risk could reduce HIV incidence 73. For moderate burden settings, mathematical models can project the incremental benefits and costs of PrEP in addition to, and, in comparison to other HIV prevention interventions. Data on real world uptake and use are needed here to guide program delivery. Integration of PrEP into platforms that provide health care, such as family planning services and STI testing, can share administrative costs thus decreasing the cost of PrEP delivery. Routine programme evaluation, including costing, can be used to estimate the cost of streamlined PrEP delivery within and outside facilities 74, 75.

In summary, oral PrEP is a novel HIV prevention strategy which has high efficacy when used consistently around the time of HIV exposure and about which we are learning about successful delivery. The early lessons from oral PrEP demonstration projects among African AGYW are relevant to longer‐acting and less adherence‐dependent HIV prevention formulations which are in development, including the dapivirine vaginal ring, injectable cabotegravir, and antiretroviral implants. Longer‐acting PrEP formulations will be simpler for women to use but will also require demand creation, goal setting, risk assessment, adherence support, and simple delivery models in order to achieve the coverage needed to have a public health impact. As with the contraception field, it is important to provide women with a choice of products that meet their reproductive and HIV prevention needs and learn how to support their choice and facilitate their uptake and persistence during periods of high risk.

## Competing interests

Dr Celum received donations of Truvada from Gilead Inc for the conduct of research studies and scientific advisory fees from Merck outside the submitted work; Dr Delany‐Moretlwe has received a donation of Truvada for a research study; Dr. Baeten has received scientific advisory fees from Gilead and Merck outside the submitted work. Dr. van der Straten received non‐financial support from Gilead sciences outside the submitted work. Dr. Bekker reports non‐financial support from Gilead and Merck outside the submitted work. Dr Hosek has received donations of Truvada for research studies.

## Authors’ contributions

CC designed and wrote the paper. SD‐M, JB, AV‐S, SH, EB, MM, RB and LGB contributed data, drafted sections of the manuscript and reviewed the final manuscript.
